# Epithelioid Angiosarcoma of the Urinary Bladder After Brachytherapy for Prostatic Carcinoma: A Case Report and Literature Review

**DOI:** 10.7759/cureus.77414

**Published:** 2025-01-14

**Authors:** Ali Ramadhan

**Affiliations:** 1 Department of Pathology, Antwerp University Hospital, Edegem, BEL

**Keywords:** epitheloid angiosarcoma, histopathology and immunohistochemistry, histopathology findings, malignant vascular tumor, radiation-induced sarcoma, radiotherapy, urinary bladder

## Abstract

Angiosarcoma of the urinary bladder is an extremely uncommon entity, with only a limited number of cases reported, making its diagnosis and management particularly challenging. This case report presents a rare occurrence of angiosarcoma within the urinary bladder and discusses its clinical presentation, radiological findings, histopathological features, and possible treatment approach for this challenging malignancy.

An 84-year-old male presented at the emergency department with hematuria. Relevant history includes prostate carcinoma Gleason 7 in 2007, treated by brachytherapy. Imaging revealed bilateral hydronephrosis, urinary retention, and an irregular lesion growing through the bladder wall. Histopathology showed a highly cellular, poorly differentiated tumor composed of epithelioid cells. Immunohistochemistry was negative for epithelial, urothelial, and prostate markers. There was positivity for ERG, CD34, CD31, factor VIII, and c-myc, confirming the diagnosis of an epithelioid angiosarcoma. RNA-based sequencing did not reveal gene rearrangements. Follow-up CT showed pulmonary metastatic disease. The patient chose to undergo euthanasia six months after symptoms onset because of the decline in quality of life.

We present a rare case of angiosarcoma arising in the urinary bladder, emphasizing the importance of considering this malignant vascular tumor in the differential diagnosis of bladder masses. Accurate diagnosis through histopathological evaluation and immunohistochemical staining is crucial for appropriate management. Characteristic features include its infiltrative vascular phenotype with marked atypia and positive staining for endothelial markers. Further studies are warranted to enhance our understanding of this rare entity and establish optimal treatment strategies.

## Introduction

Most urinary bladder neoplasms are primary carcinomas. Malignant mesenchymal tumors occur frequently in the retroperitoneum, uterus, extremities, and bones, but they are rare in the urinary bladder. Angiosarcomas involving the urinary bladder are very rare. The clinical and radiological findings of bladder epithelioid angiosarcomas are similar to those of primary bladder carcinomas, making preoperative diagnosis challenging. The angiosarcomas have a predilection for the skin, soft tissues, breast, liver, spleen, and bone but are rare in the genitourinary tract. The association of angiosarcoma with long-term trauma, radiation therapy, and chronic inflammation is well known. The bladder, however, is a rare primary site; only a handful of cases have been reported in the English literature. Epithelioid angiosarcoma (EA) has, like other angiosarcomas, an endothelial cell lining with a rather cuboidal or polygonal shape, giving the cell an epitheliod-like appearance, but it should not be misdiagnosed as an adenocarcinoma [1].

To the best of our knowledge we present the 47th reported case of primary angiosarcoma of the urinary bladder in literature and the 20th of radiation-induced epithelioid subtype angiosarcomas of the bladder [2,3].

A literature review by thorough and detailed search of electronic databases PubMed, Web of Science, an online library provided by the WHO (Hinari), and Scopus was retrospectively performed until June 2024 using the following search terms: ("epithelioid angiosarcoma" or "epithelioid hemangioendothelioma" or "hemangioendothelioma") and ("urinary bladder" or "bladder"). Only articles with similar topics were considered in the review.

Angiosarcoma involving the urinary tract is a rare entity, with the majority of cases occurring in the prostate and seminal vesicles and no more than even 300 reported cases of (majority were well-differentiated) angiosarcoma involving the urinary bladder. Clinical findings and imaging studies usually correlate to a region of urogenital tract involvement. Early diagnosis, referral to a tertiary cancer center, aggressive surgical management, and multimodal therapy are important to improve patient survival outcomes [2].

Sites of origin of EA are numerous, with the deep soft tissue being the most commonly involved site, but a variety of primary sites are encountered. Interestingly, the urinary bladder has not been listed as a site of origin in recent series [4]. Histologically, angiosarcoma is characterized by spindled, polygonal, epithelioid, and primitive round cells, with expression of both vascular and endothelial antigens on immunohistochemistry, including Factor-VIII, CD31, CD34, and ERG. The ERG immunohistochemistry can especially be beneficial in distinguishing EA from sarcomatoid epitheliod carcinomas [5].

Epithelioid angiosarcoma is a rare malignant vascular neoplasm of endothelial cells, accounting for 5-20% of all angiosarcomas. The most common sites of EA besides deep or superficial soft tissues are (heart, liver, and spleen) and may arise in various other sites (skin, breast, and thoracic duct). Although EA is more common in the soft tissues and visceral thoracic sites, EA involving the urinary bladder has been sporadically reported. We aim to present a new case of EA involving the urinary bladder and summarize the characteristics of thirteen bladder EAs described in the previous reports [4,6,7].

To date, CD34, CD31, and ERG are the most frequently used endothelial markers for epithelioid angiosarcoma, as demonstrated by immunohistochemistry. These markers are useful in making the distinction between angiosarcoma, including those with epitheliod morphology, and other spindle cell tumors with epithelioid appearance [8].

The difficulty in noninvasively visualizing the bladder wall itself has stratified the task of diagnosing bladder conditions to the skills of radiologists, pathologists, and clinicians. Patterns of bladder involvement by malignancies and malignancy-mimicking conditions are useful in approaching the task of diagnosing disease in the urinary bladder. To rule out malignant disorders of the bladder, invasive studies, such as cystoscopy with biopsy or transurethral resection, aid in identifying and characterizing the diseased area. As specific patterns may suggest malignancy, it is critical for pathologists to recognize these patterns and their associated radiologic findings and correlate the imaging and histologic findings with the clinical history and any other diagnostic information available [8].

Bladder angiosarcoma imitates carcinoma, and most patients undergo radical cystectomy alone, which is not adequate for the treatment of angiosarcoma. Angiosarcoma invading adjacent organs is an indication for adjuvant chemotherapy [9]. The prognosis of epithelioid angiosarcomas is generally deemed dismal, with a reported mean survival time of 15 months [10].

## Case presentation

An 84-year-old male patient was referred to our clinic with a one-month history of frequent micturition and dysuria. The patient has a history of prostate carcinoma Gleason 7 in 2007, treated by curative brachytherapy with radiation proctitis as a sequela. Clinical examination shows a non-tender abdomen with suprapubic pain on palpation. The patient's clinical parameters were all within normal range. Urine analysis revealed increased WBCs (792/µL (normal range<18/µL)) with prominent hematuria RBCs (266024/µL (normal range<15/µL)) while PSA and creatinine were within normal limits.

**Figure 1:**
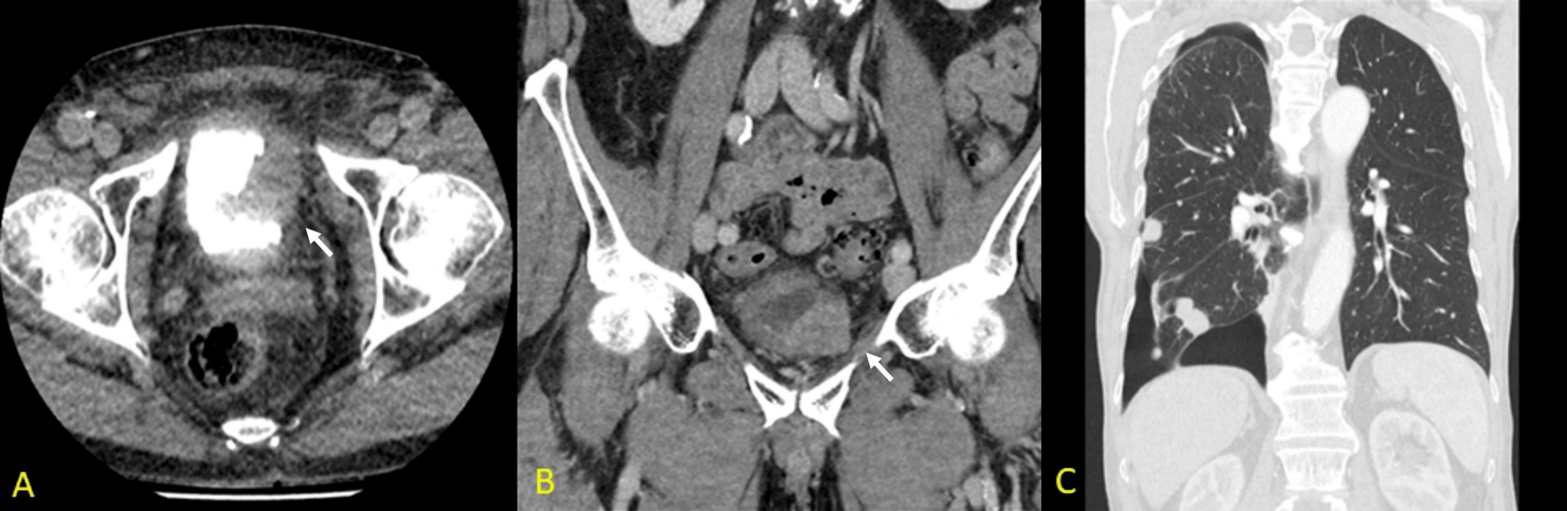
Imaging studies. (A, B) Urinary tract CT revealed an irregular, partial hyperreflective and reflective lesion growing through the bladder (white arrows). (C) A follow-up CT showing pulmonary metastasis.

Ultrasound revealed bilateral hydronephrosis and retention of intravesicular hyperechogenic material. Segmental thickening on the lateral side of the bladder was detected on computed tomography (CT) scans shown in Figure 1A and Figure 1B. In contrast, a mass lesion in the posterior bladder wall was detected in MRI. Rectal wall invasion could not be evaluated radiographically. A follow-up CT was performed 5 months after diagnosis and revealed multiple pulmonary metastases (Figure 1C). A transurethral resection for presumably non-muscle-invasive bladder cancer has been performed at another clinic.

**Figure 2:**
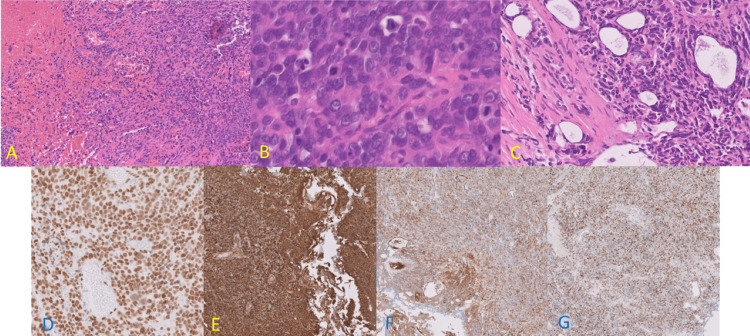
Histopathological picture. The tumor showed anastomotic vascular spaces (A), lined by epithelioid tumor cells with high mitotic activity, and cytonuclear atypia (B), muscular invasion was prominent (C), the tumor cells are positive for ERG (D), CD31 (E), factor VIII (F) and c-myc (G).

Surprisingly, the pathological examination demonstrated a tumor with irregular thick compartment septa. The tumor constitutes solid areas composed of atypical epithelioid to spindle cells. The epithelioid tumor cells had markedly pleomorphic, vesicular nuclei, evident nucleoli, and moderate cytoplasm, as shown in Figures 2A, 2B, 2C.

Immunohistochemistry of the tumor was negative for any epithelial, urothelial, or prostate markers besides basic sarcoma markers, including (CK, EMA, NKX3.1, GATA3, P63, SMA desmin, and myogenine). They were positive for ERG, CD34, CD31, factor VIII, and c-myc (Figure 2D, 2E, 2F, 2G), confirming the diagnosis of an epithelioid angiosarcoma. RNA-based sequencing did not reveal gene rearrangements. Morphologically and immunohistochemically, the tumor was diagnosed as epithelioid angiosarcoma (intermediate grade). The differential diagnosis included recurrence of ERG-positive prostatic carcinoma, undifferentiated carcinoma, melanoma, and malignant rhabdoid tumor. The patient was informed about the possible options for treatment, surgery, the added values of radiotherapy and chemotherapy, and the rapid course and mortality risk. The patient was subsequently treated with primary radiotherapy. After five months, follow-up CT (Figure 1C) showed progressive pulmonary metastasis. Due to the decline in quality of life, the patient chose to undergo euthanasia six months after the onset of symptoms.

## Discussion

Primary angiosarcoma of the bladder is extremely rare, with a mostly unknown etiology. The male-to-female ratio is about 5:1, with the average age of diagnosis being 64 years, although a single case at the age of 20 has been described [11]. Risk factors include smoking and pelvic radiation. The most common symptoms at presentation are painless hematuria and lower urinary tract symptoms, followed by pelvic pain [1]. Few cases have been reported where cutaneous metastatic nodules were the first sign [12,13]. 

Angiosarcomas of the bladder follow a highly aggressive course with widespread metastasis, mostly to lymph nodes, peritoneum, liver, and lung. Therapy can include partial or radical resection with adjuvant radiotherapy and/or chemotherapy active in sarcomas. Due to its rarity, no standardized treatment plan exists, and treatment is decided on an individual basis. Clinical outcomes are often poor despite extensive therapy, with an average survival of less than one year [1].

Angiosarcomas usually express vascular and endothelial markers, including ERG, CD31, CD34, and factor VIII, with CD31 being the most sensitive [8]. An important pitfall is the fact that epithelioid angiosarcoma may be positive for keratins and EMA, prompting suspicion of a carcinoma. In the bladder, the most prominent differential diagnoses are hemangioma, Kaposi sarcoma, high-grade urothelial carcinomas, and urothelial carcinomas with sarcomatoid differentiation [14]. Radiation-associated angiosarcomas in other sites are associated with a *MYC*-gene amplification. However, in nine reported cases of the bladder, only one case reported harboring *MYC*-gene amplification with fluorescence in situ hybridization [15].

To the best of our knowledge, this is the 47th reported case of primary angiosarcoma of the urinary bladder and the 20th radiation-induced epithelioid subtype angiosarcoma of the bladder [2,3].

## Conclusions

This case report discusses the presentation, histopathological features, and treatment approach in a challenging case of epithelioid angiosarcoma of the urinary bladder arising years after radiation treatment for prostatic carcinoma. The importance of immunohistochemistry in differentiating this highly aggressive tumor from its lookalikes is addressed. Only few similar cases have been previously reported in the literature. 

## References

[1] Gerbaud F, Ingels A, Ferlicot S, Irani J (2017). Angiosarcoma of the bladder: review of the literature and discussion about a clinical case. Urol Case Rep.

[2] Williams S, Romaguera R, Kava B (2008). Angiosarcoma of the bladder: case report and review of the literature. Sci World Journ.

[3] Pierce D, Connelly ZM, Boyd F, Heinsimer K (2023). De novo angiosarcoma of the bladder: a case report. Curr Prob Cancer: Case Rep.

[4] Hart J, Mandavilli S (2011). Epithelioid angiosarcoma: a brief diagnostic review and differential diagnosis. Arch Pathol Lab Med.

[5] Gaballah AH, Jensen CT, Palmquist S, Pickhardt PJ, Duran A, Broering G, Elsayes KM (2017). Angiosarcoma: clinical and imaging features from head to toe. Br J Radiol.

[6] Sbaraglia M, Bellan E, Mentzel T, Dei Tos AP (2021). The contribution of Juan Rosai to the pathology of soft tissue tumors. Pathologica.

[7] Liu H, Huang X, Chen H, Wang X, Chen L (2014). Epithelioid angiosarcoma of the kidney: a case report and literature review. Case Rep Oncol Med.

[8] Hu X, Li G, Wu S (2022). Advances in diagnosis and therapy for bladder cancer. Cancers (Basel).

[9] Bahouth Z, Masarwa I, Halachmi S, Nativ O (2015). Primary angiosarcoma of urinary bladder: 13th reported patient. Case Rep Oncol Med.

[10] Cito G, Santi R, Gemma L, Galli IC, Li Marzi V, Serni S, Nesi G (2021). Angiosarcoma of the urinary bladder following radiotherapy: report of a case and review of the literature. Medicina (Kaunas).

[11] Beyazal M, Pirinççi N, Yavuz A, Özkaçmaz S, Bulut G (2014). Computed tomography and magnetic resonance imaging findings of primary bladder angiosarcoma: a case report. Clin Imaging.

[12] Schwartz RA, Kardashian JF, McNutt NS, Crain WR, Welch KL, Choy SH (1983). Cutaneous angiosarcoma resembling anaplastic Kaposi's sarcoma in a homosexual man. Cancer.

[13] Morgan MA, Moutos DM, Pippitt CH Jr, Suda RR, Smith JJ, Thurnau GR (1989). Vaginal and bladder angiosarcoma after therapeutic irradiation. South Med J.

[14] Wang G, Black PC, Skinnider BF, Hayes MM, Jones EC (2016). Post-radiation epithelioid angiosarcoma of the urinary bladder and prostate. Can Urol Assoc J.

[15] Panwar V, Tintle SJ, Koorse Germans S, Koduru P, Jia L (2022). MYC amplification in epithelioid angiosarcoma of the urinary bladder and prostate following prostate radiotherapy: a case report with a novel molecular alteration. Int J Surg Pathol.

